# Development of small molecules promoting tau degradation in Alzheimer disease

**DOI:** 10.1002/alz70859_106337

**Published:** 2025-12-26

**Authors:** Bengt Winblad, Lisha Wang, Banesh Sooram, Thomai Mouskeftara, Rajnish Kumar, Sophia Schedin Weiss, Lars O Tjernberg

**Affiliations:** ^1^ Division of Neurogeriatrics, Karolinska Institutet, Solna, Stockholm Sweden; ^2^ Theme Inflammation and Aging, Karolinska University Hospital, Huddinge, Stockholm Sweden; ^3^ Indian Institute of Technology, Varanasi India; ^4^ Karolinska Institutet, Solna, Stockholm Sweden

## Abstract

**Background:**

Intraneuronal accumulation of tau aggregates is one of the hallmarks of AD brain pathology. Lowered efficiency of degradation pathways, such as ubiquitin proteasome system, further exacerbates this accumulation. Proteolysis‐Targeting Chimeras (PROTACs) are hetero‐bifunctional molecules that can bring E3 ligase into the vicinity of the protein of interest, leading to protein ubiquitination followed by proteasomal degradation. Since 2016, PROTACs have been applied to resolve tau pathologies. The exploration of these PROTACs is still at the early stage. In this study, we aim to develop novel small‐molecule PROTACs to promote tau degradation for AD treatment.

**Methods:**

We manually generated an in silico library of novel PROTACs and modelled their ability to form the ternary complex (tau‐PROTACs‐E3 ligase) using targeted protein degradation tools on the dedicated platform – Molecular Operating Environment. Pharmacokinetic properties were predicted by in‐silico web server – Deep‐PK. The stability and membrane permeability of top‐ranked PROTACs are now evaluated by molecular dynamics simulations. Meanwhile, we established different cell models to evaluate PROTACs’ effects on tau pathology: human neuroblastoma SH‐SY5Y cells with human tau overexpression and mouse embryonic primary neurons with tau pathology. Now the effects of our synthesized PROTACs on total tau and phospho‐tau levels are studied by using western blot and immunofluorescence staining. Underlying mechanisms on tau degradation are investigated by using proteasome/autophagy inhibitors and co‐immunoprecipitation.

**Results:**

Within around 700 new designed PROTACs, we found by computational modelling more than 25 PROTACs that were predicted to have a better ability to form ternary complexes than the previously reported PROTACs. Pharmacokinetic predictions suggested that several PROTACs should have good oral bioavailability, brain permeability and low toxicity. Some of the selected PROTACs showed good stability during molecular dynamics simulations. The membrane permeability is currently being assessed. In primary neurons, Aβ42 treatment induces tau hyperphosphorylation, and the reported PROTACs (eg. C004019) can reverse tau hyperphosphorylation to normal levels. The evaluation of our new PROTACs in cell models are being performed according to methods listed above.

**Conclusions:**

The development of small‐molecule PROTACs may bring breakthrough medicines for AD and other neurodegenerative diseases.

